# Progressive Changes in Household Food Safety Handling Knowledge and Behaviors Associated with a Continuous Tracing Intervention Among Rural Residents in China

**DOI:** 10.3390/foods15142487

**Published:** 2026-07-14

**Authors:** Zaidi Guo, Li Bai

**Affiliations:** 1 School of Management, Nanjing University of Posts and Telecommunications, Nanjing 210023, China; 2 College of Biological and Agricultural Engineering, Jilin University, Changchun 130022, China

**Keywords:** household food safety, food handling behavior, rural residents, tracing intervention, repeated measures, food safety education

## Abstract

Household food safety refers to consumer practices that reduce microbial contamination, cross-contamination, temperature abuse, and foodborne disease risks during food storage, preparation, cooking, and leftover handling. Rural households may be particularly vulnerable because of limited access to standardized food safety education and the persistence of experience-based practices. This study examined progressive changes in household food safety handling knowledge and behaviors associated with cumulative exposure to a continuous tracing intervention among rural residents in China. A two-month quasi-experimental repeated-measures study without a non-intervention control group was conducted in rural households in Liaoning, Jilin, and Heilongjiang provinces. A total of 139 households completed all intervention activities and four assessment waves, yielding 556 valid questionnaires. The intervention consisted of 10 rounds of paper-based and video-based materials targeting food safety knowledge, risk awareness, and self-efficacy. Outcomes were measured at baseline, after four intervention rounds, after eight rounds, and after ten rounds. Food safety behavior scores increased from 9.76 at baseline to 11.66, 13.33, and 14.72 after successive intervention stages. Knowledge scores increased from 13.71 to 17.35, 19.34, and 21.43, respectively. Pairwise comparisons showed significant differences between all assessment waves for both outcomes. Analyses accounting for the repeated-measures structure showed a positive association between food safety knowledge and self-reported handling behavior. Both online and offline delivery channels were associated with improvements in knowledge and behavior, while between-channel differences did not appear to be the primary driver of the observed changes. However, causal interpretation should be cautious because the study lacked a non-intervention control group, and testing effects, maturation, seasonal influences, and social desirability cannot be ruled out.

## 1. Introduction

Food safety is commonly discussed in relation to agricultural production, food processing, transportation, retailing, and regulatory control. However, the household stage is also a decisive point in the farm-to-table food safety chain. Consumers often assume that buying food from a trusted source is sufficient to ensure safety, but safe food can become unsafe at home if it is transported improperly, stored at unsuitable temperatures, thawed repeatedly, cross-contaminated during preparation, undercooked, or handled incorrectly after cooking. In this sense, consumers are not only passive recipients of food safety governance but also active final gatekeepers whose practices can either preserve or undermine the safety of food before consumption [1,2].

In this study, household food safety refers to safe consumer handling practices that reduce microbial contamination, cross-contamination, temperature abuse, and foodborne disease risks during purchasing, storage, thawing, preparation, cooking, and leftover handling. These risks may involve raw meat, poultry, seafood, eggs, dairy products, vegetables, fruits, and cooked leftovers, each of which requires appropriate storage, separation, thawing, preparation, or reheating practices [3]. The use of refrigerator and cooking thermometers has also been examined as a practical approach to supporting appropriate temperature control in household food handling [4].

The household stage is particularly important in rural communities. Rural households may have strong practical knowledge of food production and cooking, but this does not necessarily mean that their daily food handling practices meet modern food safety recommendations. In many rural households, food safety knowledge may be transmitted through family experience rather than standardized education. Rural households may also differ in their access to standardized food safety information and in the conditions under which food is stored and prepared. These contextual considerations create a need for food safety interventions that are accessible, repeated, and adapted to rural household conditions [5].

Previous food safety interventions have used lectures, printed materials, face-to-face training, and related educational approaches [6]. Qualitative evidence has identified practical, motivational, and contextual barriers to safe food handling [7]. Theory-informed intervention and behavioral studies have linked food-handling practices to behavioral intention, knowledge, perceived risk, self-efficacy, and household context [8,9,10,11,12]. Therefore, repeated and interactive intervention may be needed to reinforce key messages and support safer practices over time. Existing studies have documented gaps between self-reported and observed household food safety behaviors [13]. Intervention studies have also examined strategies for improving household food-handling behavior [14].

Recent high-quality evidence further supports the need to move beyond knowledge-only food safety education. Reviews of microbial food safety education in China and global intervention studies indicate that consumer-oriented interventions should address not only knowledge, but also risk perception, attitudes, self-efficacy, habits, and the practical context in which food is handled [15,16]. Studies conducted during and after the COVID-19 pandemic also suggest that household food safety behavior is shaped by perceived risk, trusted information sources, and psychological predictors from extended behavioral models [17,18]. In addition, recent studies have examined several consumer food safety education and behavior-change approaches. Self-efficacy-based interventions [19], structural KAP modelling [20], and game- or video-based online interventions [21] have provided evidence for improving or explaining safe food-handling practices. Other studies have highlighted the limitations of knowledge-only approaches and identified gaps in actual practices among food handlers, higher-risk groups, and other population-specific samples [22,23,24]. Evidence from media campaigns, behavior-change techniques, and online food-safety education further supports the value of repeated, targeted, and interactive delivery [25,26,27]. Recent studies from China, including nudge-based intervention research [28] and national household outbreak attribution [29], further highlight the importance of consumer-side behavior in preventing domestic foodborne risks.

This study aimed to examine progressive changes in household food safety handling knowledge and self-reported behavior across cumulative intervention waves among rural residents in China. Related findings from this research program have been reported previously in the author’s doctoral thesis and in a publication comparing online and offline intervention effects [30,31]. Building on those earlier reports, the present manuscript focuses on progressive changes across repeated assessment waves and on the knowledge–behavior association under cumulative intervention exposure.

The objectives of this study were: (1) to evaluate whether household food safety handling knowledge and behavior scores improved across repeated intervention waves; (2) to examine whether the scores showed a progressive pattern from baseline through four, eight, and ten intervention rounds; (3) to analyze the association between food safety knowledge and behavior scores; and (4) to discuss implications for rural food safety education and household-level intervention design.

## 2. Materials and Methods

### 2.1. Study Design and Setting

This study used a quasi-experimental repeated-measures design to examine progressive changes in household food safety handling knowledge and behaviors associated with a continuous tracing intervention. The intervention was conducted over approximately two months from July to September 2019 in rural communities in Northeast China. Participants were recruited from three provinces: Liaoning, Jilin, and Heilongjiang. The study design included 10 intervention rounds and four assessment waves: before the intervention (T0), after four intervention rounds (T1), after eight intervention rounds (T2), and after ten intervention rounds (T3).

The study was designed to reflect realistic rural education conditions rather than a highly controlled laboratory setting. Intervention materials were delivered through both offline and online channels, but the present analysis focuses on cumulative intervention exposure. The study therefore evaluates whether repeated contact with structured materials and follow-up could generate progressive improvements in household food safety handling knowledge and behavior.

### 2.2. Participants and Recruitment

A total of 139 rural households completed the full intervention process and all four assessment waves, generating 556 valid questionnaires. The sample included 42 households from Liaoning Province, 52 from Jilin Province, and 45 from Heilongjiang Province. Participants were household members who were involved in food purchasing, cooking, or daily food handling. This criterion was important because the intervention targeted actual household food handlers rather than general residents without food preparation responsibilities.

The final sample was predominantly female, reflecting the role of women in household food purchasing and meal preparation in many rural settings. Among the 139 participants, 49 were male and 90 were female. The largest age groups were 35–44 years and 45–54 years, and most participants had completed primary or junior high school education. More than 90% of participants reported that they frequently cooked or purchased food at home, indicating that the sample was appropriate for evaluating household food safety handling practices.

### 2.3. Intervention Design

The intervention was developed around three behavioral targets: food safety self-protection knowledge, food safety awareness, and self-efficacy. These targets were selected because household food safety practices depend on factual understanding, risk perception, and confidence in performing recommended behaviors. The intervention materials were selected and adapted from national official websites and authoritative food safety education resources. Specifically, the kitchen food safety guidance materials were compiled with reference to materials from the Chinese Center for Disease Control and Prevention (China CDC) and the World Health Organization’s Five Keys to Safer Food Manual [2]. The educational videos on foodborne pathogens, including pathogenic *Escherichia coli* and *Salmonella*, and the video on food label interpretation were obtained from or adapted from resources released by the China National Center for Food Safety Risk Assessment (CFSA). Before implementation, an expert group reviewed the intervention design, intervention materials, and feasibility of implementation. The materials were further adapted to the rural household context and focused on three intervention targets: household food safety handling knowledge, food safety awareness, and self-efficacy.

The same core materials were used in the online and offline groups; the channels differed only in delivery mode and feedback procedure. The intervention included printed or electronic leaflets and short educational videos covering foodborne hazards, safe handling of meat, vegetables, and fruits, food labels, storage and thawing, kitchen hygiene, foodborne pathogens, and leftover handling.

In the online group, materials were distributed through WeChat groups, and participants confirmed completion by replying under the article or sending “Read” or “Watched”. In the offline group, printed materials were delivered face-to-face, and videos were watched together with investigators. Brief semi-structured question-and-answer discussions were used to check understanding and answer questions. Each contact was conducted approximately every three days and lasted about 20 min.

The 10 intervention rounds covered four household food safety domains: purchasing, storage and thawing, food preparation, and leftover handling. The main intervention stages and behavioral targets are summarized in Table 1.

The selection of intervention targets was broadly informed by health behavior theories, including the Theory of Planned Behavior [32], Social Cognitive Theory [33], the Health Belief Model [34], and Protection Motivation Theory [35]. The use of behavioral theory in intervention development was also consistent with methodological recommendations for designing and testing health behavior interventions [36]. Food safety knowledge, risk awareness, and self-efficacy are related to constructs such as perceived risk, cues to action, behavioral intention, and confidence in performing recommended behaviors. However, the intervention was not fully mapped to a single theoretical framework, and the study did not directly measure mediating constructs such as perceived risk, cue salience, self-efficacy, or habit strength. Therefore, the theoretical interpretation should be regarded as mechanism-consistent rather than mechanism-confirming.

### 2.4. Outcome Measures

Two primary outcomes were assessed: household food safety handling behavior score and household food safety knowledge score. The same questionnaire and scoring rules were used at all four assessment waves, baseline (T0), after four intervention rounds (T1), after eight rounds (T2), and after ten rounds (T3), to ensure comparability of within-participant changes over time.

The food safety knowledge section contained 36 items covering food purchasing, storage and thawing, food preparation, kitchen hygiene, leftover handling, and foodborne disease knowledge. Each correct answer was coded as 1, whereas an incorrect or “do not know” answer was coded as 0, yielding a possible score ranging from 0 to 36. Higher scores indicated greater household food safety knowledge. Example items included questions on food label interpretation, safe transportation of raw meat and seafood, appropriate thawing, refrigerator temperature, handwashing, separation of raw and cooked foods, and safe handling of leftovers.

The food safety behavior section contained 27 questions, of which 20 scored questions were used to calculate the behavior score. The total behavior score ranged from 0 to 22, with higher scores indicating more standardized and safer household food handling behavior. The remaining seven behavior questions were used for descriptive purposes and were not included in the behavior score.

The questionnaire was developed with reference to previous food safety and food-handling studies [1,2,20]. To evaluate content validity, six experts in consumer food safety behavior, food economics, and consumer health education reviewed both the knowledge and behavior items through a focus group discussion and assessed their relevance and representativeness. The questionnaire was revised according to expert comments. A pre-survey was then conducted among 48 rural participants in Jilin Province to test the clarity, feasibility, and reliability of the knowledge and behavior sections. The pre-survey data showed acceptable internal consistency, with an overall Cronbach’s alpha coefficient of 0.80.

The complete questionnaire is provided in Supplementary Table S1.

### 2.5. Statistical Analysis

To examine progressive changes across cumulative intervention exposure, scores were compared across four assessment waves: baseline (T0), after four intervention rounds (T1), after eight rounds (T2), and after ten rounds (T3). For household food safety handling behavior scores, normality tests indicated that the repeated measurements did not meet the normality assumption. Therefore, the Friedman test was used to assess overall differences across repeated waves, followed by pairwise comparisons. When the homogeneity of variance assumption was not met, Tamhane’s post-hoc test was used. These analyses were conducted using IBM SPSS Statistics version 25.0.

As a robustness analysis, linear mixed-effects models were fitted to account for the non-independence of repeated observations within participants. Assessment wave was included as a fixed effect, and participant ID was included as a random intercept. Separate mixed-effects models were fitted for food safety handling behavior score and food safety knowledge score. The mixed-effects analyses were conducted using Python statsmodels version 0.14.6. A two-sided *p* value of <0.05 was considered statistically significant.

The knowledge–behavior association was further examined using mixed-effects models. In addition, a within-person and between-person decomposition was conducted by calculating each participant’s mean knowledge score across the four waves and the deviation of each wave-specific knowledge score from that participant’s own mean. Both components were entered into a mixed-effects model predicting behavior score, with assessment wave included as a fixed effect and participant ID as a random intercept.

## 3. Results

### 3.1. Participant Characteristics

A total of 139 rural households completed the intervention. Participants were distributed across Liaoning (*n* = 42), Jilin (*n* = 52), and Heilongjiang (*n* = 45). Women accounted for 64.7% of the sample, and most participants were between 35 and 54 years old. Educational attainment was generally low to moderate: 25.2% had completed primary school and 55.4% had completed junior high school. Most participants were married, and the most common annual household income category was 36,000–60,000 RMB. These characteristics are consistent with the study objective of targeting rural household food handlers who may benefit from practical food safety education. The demographic characteristics of the participants are summarized in Table 2.

### 3.2. Overall Progressive Pattern in Behavior and Knowledge Scores

Both outcome indicators increased steadily across the four assessment waves. The mean food safety handling behavior score increased from 9.76 at baseline to 11.66 after four intervention rounds, 13.33 after eight intervention rounds, and 14.72 after ten intervention rounds. The total increase from baseline to the final assessment was 4.96 points. The mean food safety knowledge score increased from 13.71 at baseline to 17.35 after four intervention rounds, 19.34 after eight intervention rounds, and 21.43 after ten intervention rounds, with a total increase of 7.73 points.

**Figure 1:**
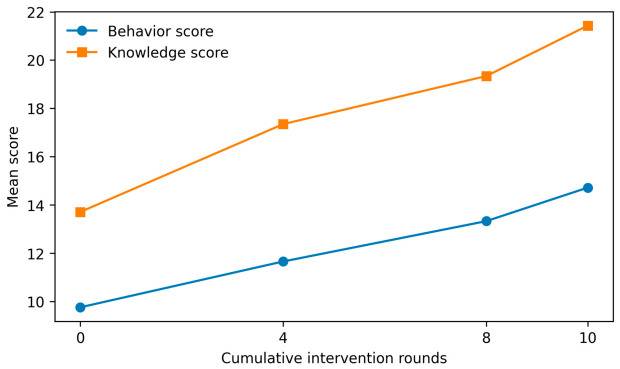
Mean household food safety handling behavior and knowledge scores across cumulative intervention exposure.

The increments between adjacent waves were 1.90 points from T0 to T1, 1.67 points from T1 to T2, and 1.39 points from T2 to T3 for behavior scores, and 3.65 points, 1.99 points, and 2.09 points for knowledge scores, respectively. These results show a progressive increase in both behavior and knowledge scores across cumulative intervention exposure, although the increases were not uniform across waves. Descriptive statistics for the two outcomes are presented in Table 3, and their progressive patterns are illustrated in Figure 1.

### 3.3. Pairwise Comparisons Across Assessment Waves

The overall repeated-measure comparison indicated significant differences in behavior scores across the four assessment waves. Because the behavior score did not meet the normality assumption, a Friedman test showed significant differences across time points (*p* < 0.001). Pairwise comparisons further indicated that scores were significantly higher at each subsequent assessment wave. The behavior score after four rounds was significantly higher than the baseline score; the score after eight rounds was significantly higher than that after four rounds; and the final score after ten rounds was significantly higher than that after eight rounds.

Knowledge scores also differed significantly between all assessment waves. Pairwise comparisons showed that knowledge improved significantly from baseline to four rounds, from four to eight rounds, and from eight to ten rounds. These findings support the presence of a cumulative intervention effect. At the same time, the slope of improvement was not constant. Knowledge improved most strongly during the initial intervention stage, while behavior improved more gradually. This difference is theoretically plausible because knowledge acquisition may occur faster than behavioral standardization, whereas behavior change requires translation of knowledge into repeated household practice. The pairwise comparison results are summarized in Table 4.

### 3.4. Robustness Analysis Using Linear Mixed-Effects Models

As a robustness analysis, linear mixed-effects models were fitted to account for the non-independence of repeated observations within participants. Participant ID was included as a random intercept, and assessment wave was included as a fixed effect. Separate models were fitted for household food safety handling behavior score and food safety knowledge score.

The mixed-effects results were consistent with the main repeated-measures analysis. Compared with baseline, behavior scores were significantly higher after four rounds (β = 1.90, SE = 0.30, 95% CI: 1.31 to 2.48, *p* < 0.001), eight rounds (β = 3.57, SE = 0.30, 95% CI: 2.98 to 4.15, *p* < 0.001), and ten rounds (β = 4.96, SE = 0.30, 95% CI: 4.37 to 5.54, *p* < 0.001). Knowledge scores were also significantly higher after four rounds (β = 3.65, SE = 0.43, 95% CI: 2.81 to 4.48, *p* < 0.001), eight rounds (β = 5.63, SE = 0.43, 95% CI: 4.80 to 6.47, *p* < 0.001), and ten rounds (β = 7.73, SE = 0.43, 95% CI: 6.89 to 8.56, *p* < 0.001).

These results indicate that the progressive increases in behavior and knowledge scores remained evident after accounting for the repeated-measures structure of the data. However, because the study did not include a non-intervention control group, the mixed-effects results should be interpreted as robustness evidence for progressive score changes rather than as definitive causal evidence of intervention effects.

### 3.5. Association Between Knowledge and Behavior

The knowledge–behavior association was reanalyzed using mixed-effects models to account for the repeated-measures structure. In a model with participant ID as a random intercept, food safety knowledge score was positively associated with food safety behavior score (β = 0.441, SE = 0.022, 95% CI: 0.398 to 0.485, *p* < 0.001). After adjusting for assessment wave, the association remained significant (β = 0.308, SE = 0.027, 95% CI: 0.256 to 0.360, *p* < 0.001).

A within-person and between-person decomposition was further conducted. The within-person association was positive and significant (β = 0.330, SE = 0.030, 95% CI: 0.271 to 0.389, *p* < 0.001), indicating that participants tended to report safer food handling behavior at waves when their knowledge score was higher than their own average knowledge score. The between-person association was also positive and significant (β = 0.238, SE = 0.054, 95% CI: 0.131 to 0.345, *p* < 0.001), indicating that participants with higher average knowledge scores also tended to report safer average behavior.

For descriptive comparability with the original doctoral analysis, the pooled Pearson correlation is mentioned only as an exploratory descriptive result and should be interpreted cautiously because the 556 observations included repeated measurements from 139 participants and therefore did not represent 556 independent observational units.

### 3.6. Online and Offline Intervention Channels as Supplementary Evidence

The intervention was delivered through two channels: offline face-to-face delivery and online delivery through WeChat. In the offline group, 65 participants completed all assessments. Behavior scores increased from 10.22 at baseline to 15.26 after ten rounds, and knowledge scores increased from 13.89 to 22.00. In the online group, 74 participants completed all assessments. Behavior scores increased from 9.37 to 14.24, and knowledge scores increased from 13.54 to 20.93.

Both channels were therefore associated with meaningful improvement. No significant difference was observed between the online and offline groups in the magnitude of pre–post change. This finding suggests that for rural household food safety education, repeated intervention exposure may be more important than delivery channel. From a practical perspective, online delivery may provide a lower-cost option for geographically dispersed rural areas, while offline delivery may remain useful where digital literacy or internet access is limited. Outcome scores by intervention delivery channel are presented in Table 5.

## 4. Discussion

### 4.1. Principal Findings

This study found that a continuous tracing intervention was associated with progressive improvement in household food safety handling knowledge and behaviors among rural residents in China. Both outcomes increased after four, eight, and ten intervention rounds, and all pairwise comparisons were statistically significant. The findings indicate that repeated education and follow-up can produce cumulative gains in rural household food safety. Importantly, the intervention did not simply produce a one-time improvement immediately after exposure; scores continued to increase after additional rounds, suggesting a progressive pattern across cumulative exposure.

These findings should be interpreted in the context of previous research on consumer-side food safety and household food handling. Household food safety practices, including separation of raw and cooked foods, safe storage, proper thawing, sufficient reheating, hand hygiene, and safe handling of leftovers, have been identified as important factors in reducing domestic foodborne risks [1,2,13]. Previous studies have also shown that consumers’ food safety knowledge and self-reported practices are often uneven, and that unsafe handling may persist even when general awareness of food safety is present [15,16].

The magnitude of improvement differed between knowledge and behavior. Knowledge increased rapidly during the initial intervention stage and continued to improve with further exposure. Behavior also improved steadily but more gradually. This difference is consistent with behavioral theory. Knowledge can change relatively quickly when participants receive clear information, but behavior depends on daily opportunities, household routines, perceived barriers, and confidence. For example, a participant may quickly learn the recommended refrigerator temperature or the importance of separating raw and cooked food, but regular application requires habit formation and repeated cues during actual food preparation. This is consistent with previous studies showing that food safety knowledge does not always translate directly into safe handling behavior [22].

### 4.2. Potential Mechanisms of Progressive Change

The observed progressive changes are consistent with several plausible theoretical mechanisms, although these mechanisms were not directly tested in the present study. First, repeated exposure may increase message retention. A single lecture or leaflet may be forgotten, especially when households are busy or when the recommended behavior is not immediately practiced. Repeated contact gives participants multiple opportunities to receive, process, and remember the information. This interpretation is consistent with food safety intervention studies emphasizing repeated exposure, self-efficacy, and behaviorally targeted reinforcement [19,21,25,26,28].

Second, repeated intervention may provide cues to action. In the Health Belief Model, cues to action are important triggers that help individuals translate perceived risk and perceived benefits into behavior. In this study, each intervention round may have acted as a reminder to reconsider daily food handling practices. However, cue salience and perceived risk were not directly measured; therefore, this explanation should be interpreted as theoretically plausible rather than empirically confirmed.

Third, continuous tracing may be consistent with the role of self-efficacy emphasized in Social Cognitive Theory. Many rural residents may know that certain practices are recommended but may not feel confident in applying them consistently. The intervention encouraged feedback, questions, and interaction with investigators, which may have reduced uncertainty and made safe practices appear more manageable. However, self-efficacy was not directly measured in this study.

Fourth, repeated reinforcement may be consistent with habit formation. Food handling practices often involve multiple family members and are shaped by routine. When safe handling messages are delivered repeatedly, they may gradually become part of everyday household conversation and practice. Nevertheless, habit strength was not assessed directly.

Accordingly, these explanations should be regarded as mechanism-consistent interpretations rather than mechanism-confirming evidence. The present study measured food safety knowledge and self-reported behavior scores, but did not directly measure mediating constructs such as perceived risk, self-efficacy, cue salience, or habit strength. Therefore, future studies should measure these intermediate constructs and test the proposed pathways using longitudinal mediation analysis or structural equation modeling.

### 4.3. Implications for Rural Food Safety Education

The findings have practical implications for rural food safety education. First, rural food safety programs should move beyond one-off publicity activities. One-time education may be useful for awareness raising, but household food safety behavior requires repeated reinforcement. A feasible program could deliver short messages weekly or biweekly, combining printed materials, short videos, household checklists, and interactive question-and-answer sessions. The current findings suggest that even brief repeated interventions can produce measurable gains.

Second, intervention materials should be practical and stage-specific. Rural residents need clear guidance on what to do during purchasing, storage, thawing, preparation, cooking, and leftover handling. General warnings about food safety may be less effective than concrete instructions, such as reading the shelf-life label, avoiding repeated thawing, separating raw and cooked foods, washing hands before preparing food, and reheating leftovers thoroughly. Materials should use simple language, visual examples, and culturally familiar scenarios.

Third, online and offline channels can be combined. The lack of a strong channel difference suggests that the same core content can be delivered through different formats. For villages with good smartphone use and active WeChat groups, online materials can reduce cost and allow repeated reminders. For older residents or communities with limited digital access, offline sessions remain important. A hybrid model may be particularly suitable: offline sessions can build trust and introduce key concepts, while online messages can provide follow-up reminders and reinforcement.

Fourth, local institutions can play a role in sustaining intervention. Village committees, township health centers, agricultural extension workers, schools, and local food safety regulators may all contribute to a rural food safety education network. The intervention model can be embedded into routine public health education, rural revitalization programs, or community food safety campaigns. Because foodborne illness prevention at the household level is less visible than regulatory inspection, local governments and public health agencies need simple and scalable tools for reaching households.

### 4.4. Comparison with Previous Food Safety Intervention Studies

Previous consumer food safety intervention studies have used several types of educational approaches, including one-time education, printed food safety materials, face-to-face training, online food safety education, video- or game-based interventions, media campaigns, nudge-based interventions, and theory-informed behavior-change strategies [5,6,8,19,21,25,26,28]. Reviews of microbial food safety education and consumer-oriented food safety interventions suggest that education can improve food safety knowledge and awareness, but changes in actual food handling behavior are often less consistent, especially when interventions rely only on one-way information delivery or do not address motivational, contextual, and habitual barriers [15,16,27]. This limitation is also consistent with critiques of the knowledge–attitude–practice (KAP) model, which suggest that knowledge alone does not necessarily translate into safe food handling behavior [22].

The present findings are consistent with studies showing that interactive, practice-oriented, and behaviorally targeted food safety education may be more useful than the transmission of knowledge alone. Game- and video-based online interventions, long-term media campaigns, behavior-change-technique interventions, self-efficacy-oriented interventions, and nudge-based interventions suggest that food safety behavior change may require repeated exposure, practical cues, self-efficacy enhancement, and reinforcement over time [19,21,25,26,28]. In the present study, both behavior and knowledge scores continued to increase across four, eight, and ten intervention rounds. This pattern suggests that repeated reinforcement may help participants retain food safety messages and apply them to daily household food handling practices.

The present study also differs from many previous food safety intervention studies in two ways. First, it measured both household food safety knowledge and self-reported food handling behavior at multiple assessment waves, rather than relying only on a single pre–post comparison. Second, it focused on rural households in China, a context in which food safety education may face additional challenges, including variable access to standardized food safety information, reliance on experience-based practices, dispersed residence, and differences in storage or kitchen conditions. Previous studies from China have also highlighted the importance of household-level food handling behavior and consumer-side interventions for preventing domestic foodborne risks [13,14,15,28,29].

At the same time, the findings should not be interpreted as showing that more intervention is always better without limit. Although the score trajectory showed continued increases up to ten intervention rounds, the study did not determine the minimum effective intervention frequency or the point at which additional rounds no longer provide meaningful benefit. Future randomized or cluster-randomized studies should compare different intervention frequencies and durations, measure both immediate and delayed outcomes, and include objective assessments of household food handling behavior.

### 4.5. Methodological Strengths

This study has several strengths. First, it used repeated measures within the same households, allowing changes to be evaluated over time rather than relying only on cross-sectional comparisons. Second, the intervention was implemented in real rural communities, improving practical relevance. Third, the intervention covered multiple household food safety domains rather than focusing on a single behavior. Fourth, both knowledge and behavior outcomes were assessed, making it possible to examine whether knowledge improvement was associated with more standardized behavior. Fifth, the study included both online and offline delivery formats, which increases the relevance of findings for future rural education programs.

Another strength is the dose–response perspective. In educational interventions, the concept of dose is often overlooked. Researchers may report whether an intervention worked but not whether additional exposure produced additional benefit. By comparing baseline, four rounds, eight rounds, and ten rounds, this study provides evidence that repeated exposure matters. This perspective can help policymakers and practitioners design intervention schedules that are more realistic and potentially more effective.

### 4.6. Limitations

Several limitations should be acknowledged. First, the study did not include a parallel non-intervention control group. Therefore, the observed score increases cannot be definitively attributed to the intervention alone or interpreted with the same level of certainty as findings from a randomized controlled trial. Although the repeated increases across multiple assessment waves are consistent with the possibility that cumulative intervention exposure contributed to the observed changes, alternative explanations, including repeated testing effects, maturation, seasonal influences during the study period, exposure to external food safety information, and social desirability in self-reported behavior, cannot be ruled out. To reduce these risks, the study used standardized intervention materials, trained investigators, the same questionnaire and scoring rules at all assessment waves, and the same assessment schedule for all participants. Participants were informed that their responses would be used for research purposes and were encouraged to answer honestly. Nevertheless, the findings should be interpreted as progressive changes associated with cumulative intervention exposure rather than as definitive causal treatment effects. Future research should include randomized or cluster-randomized control groups and longer follow-up.

Second, the sample size was relatively small, with 139 households completing all four assessment waves. This may limit the statistical power of the analyses and the generalizability of the findings to broader rural populations.

Third, the behavior outcome was based on questionnaire responses. Self-reported food handling behavior may overestimate actual safe practices, as noted in previous food safety studies [15,16,27]. Future studies should add direct observation, kitchen audits, or objective indicators where feasible.

Fourth, the study was conducted in rural communities in Northeast China, and findings may not generalize to rural households in other regions with different food cultures, climates, income levels, or infrastructure.

Fifth, although the intervention measured outcomes after ten rounds, it did not assess long-term maintenance after the intervention stopped. Behavior change is meaningful only if it persists. Future research should include follow-up assessments after three, six, or twelve months to determine whether households maintain improved practices. Because the same core questionnaire was repeatedly administered, possible testing effects or increased familiarity with the items cannot be excluded and should be considered when interpreting the progressive score increases.

Another limitation is that the intervention was only broadly informed by health behavior theories and was not formally mapped to a single theoretical framework at the component level. The study measured knowledge and self-reported behavior outcomes, but did not directly measure mediating constructs such as perceived risk, self-efficacy, cue salience, or habit strength. Therefore, the proposed mechanisms could not be tested through mediation analysis. Future studies should directly measure these intermediate constructs and conduct mediation analysis to test the mechanisms of change.

### 4.7. Future Research Directions

Future studies should build on this work in four directions. First, researchers should test the intervention using randomized controlled or cluster-randomized designs. Villages or households could be randomly assigned to different intervention doses, enabling stronger causal inference. Second, future research should include objective behavior measurement. Direct observation of kitchen practices, temperature monitoring, and photo-based food storage assessment could reduce reliance on self-report.

Third, intervention content should be refined using behavioral theory. For example, Health Belief Model constructs such as perceived susceptibility, perceived severity, perceived benefits, perceived barriers, self-efficacy, and cues to action can be measured before and after intervention to identify which mechanisms explain behavior change. Protection Motivation Theory and the Theory of Planned Behavior could also help explain why some households translate knowledge into action while others do not. Fourth, cost-effectiveness analysis should be added. Rural public health resources are limited, and decision-makers need to know whether additional intervention rounds justify their cost.

Digital delivery deserves particular attention. Smartphones and WeChat groups are increasingly common in rural China, but digital access and digital literacy vary by age and region. A hybrid intervention model may be more scalable than either purely offline or purely online delivery. Future studies could compare different combinations of face-to-face training, short videos, reminder messages, and peer support groups to identify optimal rural implementation strategies.

## 5. Conclusions

This study found that household food safety handling knowledge and behavior scores increased progressively following cumulative exposure to a continuous tracing intervention, with significant improvements observed after four, eight, and ten intervention rounds. Knowledge and behavior were positively correlated, suggesting that improving food safety knowledge may support more standardized household practices when reinforced through repeated intervention.

The findings indicate that intervention intensity and repeated reinforcement are important for rural household food safety education. Both online and offline channels were associated with improvement, implying that flexible delivery formats can be selected according to local conditions. For rural food safety governance, the results support a shift from one-time publicity to repeated, practical, and context-sensitive household education. These findings highlight the importance of continuous, household-level food safety interventions as a practical approach to reducing unsafe food handling behaviors and strengthening consumer-side food safety risk prevention in rural communities. Future research should verify these findings using randomized designs, objective behavior measurement, long-term follow-up, and cost-effectiveness analysis.

## Figures and Tables

**Table 1:** Intervention stages and behavioral targets of the continuous tracing intervention.

Intervention Stage	Approximate Dose	Main Content	Behavioral Targets
Stage 1	Rounds 1–2	General household food safety awareness and basic purchasing/handling guidance	Risk awareness; basic knowledge; motivation to participate
Stage 2	Rounds 3–4	Safe handling of meat, vegetables, fruits, and video-based education on foodborne pathogens	Knowledge; perceived risk; safe preparation behavior
Stage 3	Rounds 5–6	Household food preparation principles and food label interpretation	Food label knowledge; food preparation behavior; self-efficacy
Stage 4	Rounds 7–8	Kitchen prevention of foodborne disease, storage, thawing, cleaning, and leftover handling	Storage/thawing behavior; kitchen hygiene; leftover handling
Stage 5	Rounds 9–10	Video on Salmonella and final food safety hot-issue leaflet	Reinforcement; awareness; confidence in safe practices

**Table 2:** Participant characteristics (*n* = 139).

Characteristic	Category	*n*	%
Province	Liaoning	42	30.2
Province	Jilin	52	37.4
Province	Heilongjiang	45	32.4
Sex	Male	49	35.3
Sex	Female	90	64.7
Age	18–24 years	18	12.9
Age	25–34 years	21	15.1
Age	35–44 years	45	32.4
Age	45–54 years	41	29.5
Age	55–64 years	10	7.2
Age	65 years and above	4	2.9
Education	Below primary school	3	2.2
Education	Primary school	35	25.2
Education	Junior high school	77	55.4
Education	High school/technical secondary school	17	12.2
Education	College or above	7	5.0
Annual household income	<12,000 RMB	14	10.1
Annual household income	12,000–36,000 RMB	39	28.1
Annual household income	36,000–60,000 RMB	42	30.2
Annual household income	60,000–84,000 RMB	20	14.4
Annual household income	>84,000 RMB	24	17.3

**Table 3:** Changes in household food safety handling behavior and knowledge scores across cumulative intervention exposure.

Outcome	Time Point	*n*	Mean	SD	SE	95% CI	Min–Max
Behavior score	Baseline	139	9.76	3.02	0.26	9.26–10.27	4–20
Behavior score	After 4 rounds	139	11.66	3.49	0.30	11.08–12.25	5–21
Behavior score	After 8 rounds	139	13.33	3.75	0.32	12.70–13.96	5–22
Behavior score	After 10 rounds	139	14.72	4.06	0.34	14.04–15.40	6–22
Knowledge score	Baseline	139	13.71	4.47	0.38	12.96–14.45	4–25
Knowledge score	After 4 rounds	139	17.35	5.05	0.43	16.51–18.20	6–31
Knowledge score	After 8 rounds	139	19.34	5.47	0.46	18.42–20.26	6–31
Knowledge score	After 10 rounds	139	21.43	5.78	0.49	20.46–22.40	6–32

**Table 4:** Pairwise comparisons of outcome scores across assessment waves.

Outcome	Comparison	Mean Difference	*p* Value	Interpretation
Behavior	T1 vs. T0	+1.90	<0.001	Improvement after initial four rounds
Behavior	T2 vs. T1	+1.67	0.001	Additional improvement after continued exposure
Behavior	T3 vs. T2	+1.39	0.020	Further improvement after final reinforcement
Behavior	T3 vs. T0	+4.96	<0.001	Total cumulative gain
Knowledge	T1 vs. T0	+3.65	<0.001	Largest early knowledge gain
Knowledge	T2 vs. T1	+1.99	0.002	Continued knowledge improvement
Knowledge	T3 vs. T2	+2.09	0.001	Further reinforcement effect
Knowledge	T3 vs. T0	+7.73	<0.001	Total cumulative gain

**Table 5:** Outcome scores by intervention delivery channel.

Channel	*n*	Outcome	Baseline	After 4 Rounds	After 8 Rounds	After 10 Rounds
Offline	65	Behavior score	10.22	12.22	13.75	15.26
Offline	65	Knowledge score	13.89	17.60	20.11	22.00
Online	74	Behavior score	9.37	11.18	12.96	14.24
Online	74	Knowledge score	13.54	17.14	18.66	20.93

## Data Availability

The data supporting the findings of this study are available from the corresponding author upon reasonable request, subject to privacy and institutional restrictions.

## Supplementary Materials

The following supporting information can be downloaded at: https://www.mdpi.com/article/10.3390/foods15142487/s1, Table S1: Household Food Safety Handling Questionnaire.
