# Supplementary material for: Progressive Changes in Household Food Safety Handling Knowledge and Behaviors Associated with a Continuous Tracing Intervention Among Rural Residents in China
Source: Foods. 2026 Jul 14;15(14):2487. doi: 10.3390/foods15142487 (PMC13407731; doi:10.3390/foods15142487)
Supplement: Supplementary file 1 [file foods-15-02487-s001.zip › foods-4351421-supplementary.pdf]

Supplementary Table S1. Household Food Safety Handling Questionnaire

Purpose: This supplementary table provides the questionnaire used to assess household food safety handling behavior and knowledge among rural residents.

• Consistency check: The behavior section contains 27 questions. Of these, 20 scored items assess household food handling practices across purchasing, storage and thawing, food preparation/kitchen hygiene, and leftover handling. The remaining 7 behavior questions are descriptive and are not included in the behavior score.

• The knowledge section contains 36 scored items. Each correct answer was coded as 1; an incorrect answer or "do not know" was coded as 0. For multiple-selection knowledge items, a score of 1 was assigned only when all required correct options were selected and no incorrect options were selected.

• The same questionnaire and scoring rules were used at all four assessment waves: baseline (T0), after four intervention rounds (T1), after eight intervention rounds (T2), and after ten intervention rounds (T3).

• Demographic questions from the field questionnaire are listed separately at the end and are not counted in the outcome item totals.

A. Household Food Safety Handling Behavior Section (27 questions; 20 scored items)

No.	Domain	English translation and response options	Scoring/status
1	Screening/descriptive	Do you often cook or buy food at home?<br>Options: A. Yes; B. No	Descriptive; not included in behavior score.
2	Descriptive	During the past year, have you had symptoms such as diarrhea, vomiting, or abdominal pain after eating unhygienic food?<br>Options: A. Yes; B. No	Descriptive; not included in behavior score.
3	Purchasing	When buying food, do you check the shelf-life or expiry date on the product package?<br>Options: A. Always; B. Sometimes; C. Never	Scored item; greater attention to shelf-life information was assigned a higher score.
4	Purchasing	When buying food, do you prefer products with an organic food label?<br>Options: A. Always prefer products with this label; B. Sometimes prefer them; C. Never prefer them	Scored item; greater attention to the label was assigned a higher score.

No.	Domain	English translation and response options	Scoring/status
5	Purchasing	When buying food, do you prefer products with a green food label?<br>Options: A. Always prefer products with this label; B. Pay little attention to whether products have this label; C. Never pay attention to this label	Scored item; greater attention to the label was assigned a higher score.
6	Purchasing	When buying food, do you prefer products with a pollution-free food label?<br>Options: A. Always prefer products with this label; B. Pay little attention to whether products have this label; C. Never pay attention to this label	Scored item; greater attention to the label was assigned a higher score.
7	Purchasing	In what order do you usually buy vegetables, fruits, raw meat, seafood, and similar foods in a supermarket?<br>Options: A. Follow the shelf order from the entrance to the checkout; B. No fixed order; C. Buy raw meat and seafood last before checkout	Scored item; buying raw meat and seafood last was treated as the safer practice.
8	Purchasing/descriptive	How often do you buy vegetables?<br>Options: A. Once a week; B. Twice a week; C. Once every two or three days; D. Every day; E. Buy only when needed	Descriptive; not included in behavior score.
9	Purchasing/descriptive	Where does your household usually buy vegetables? (Select up to three.)<br>Options: A. Large chain supermarket; B. Small or medium chain supermarket; C. Farmers' market or individual stall; D. Community convenience store; E. Organic farm, self-grown, or grown by relatives/friends; F. E-commerce platform such as WeChat merchants or public-account platforms; G. Do not buy; mostly home-grown	Descriptive; not included in behavior score.
10	Purchasing/descriptive	What kind of vegetables do you usually buy? (Select up to three.)<br>Options: A. Good appearance and no visible damage; B. Not necessarily good-looking; C. Imported rather than domestic if available; D. Relatively expensive; E. Relatively cheap; F. Discounted by the seller; G. Seasonal; H. With organic, green, or pollution-free labels; I. Do not buy; mostly home-grown	Descriptive; not included in behavior score.
11	Purchasing/descriptive	Do you often buy pickled, smoked, or grilled foods?<br>Options: A. Never; B. Rarely, about once a month; C. Not very often, about once a week; D. Often	Descriptive; not included in behavior score.
12	Purchasing/storage	If there is expired yogurt at home, would you still drink it?<br>Options: A. Yes; B. No; C. It depends; sometimes yes and sometimes no	Scored item; not drinking expired yogurt was treated as the safer practice.
13	Purchasing/descriptive	If there is a child at home, would you specially buy foods marketed for children, such as children's soy sauce or children's meat floss?<br>Options: A. Yes; B. No	Descriptive; not included in behavior score.
14	Purchasing	Would you buy food for a child if it had no food production license number?<br>Options: A. Yes; B. No; C. I have not noticed; D. I do not know what a production license number is	Scored item; not buying such food was treated as the safer practice.
15	Purchasing	Would you buy food with poor appearance if it was discounted because it was close to its expiry date?<br>Options: A. Yes; B. No; C. It depends; sometimes yes	Scored item; not buying such food was treated as the safer practice.

No.	Domain	English translation and response options	Scoring/status
16	Preparation	How do you usually wash vegetables after buying them?<br>Options: A. Soak and wash with fruit/vegetable detergent; B. Soak and wash with hot water; C. Soak and wash with cold water; D. Wash under running cold tap water; E. Wash under running warm tap water; F. Use a fruit/vegetable washing machine; G. Wipe with a cloth or paper towel; H. Do not wash if they do not look dirty	Scored item; washing under running cold water was treated as the safer practice.
17	Storage and thawing	How do you handle a large piece of raw meat after bringing it home?<br>Options: A. Put the original package directly into the freezer; B. Cut into small pieces and put into the freezer; C. Cut into small pieces, seal separately, and put into the freezer; D. Put directly into the refrigerator compartment; E. Cut into small pieces and put into the refrigerator compartment; F. Cut into small pieces, seal separately, and put into the refrigerator compartment; G. Leave at room temperature in a cool place	Scored item; cutting into small sealed portions and freezing was treated as the safer practice.
18	Storage and thawing	How do you usually thaw frozen meat taken from the freezer?<br>Options: A. Thaw in a microwave oven; B. Soak directly in cold water; C. Seal the package and soak in cold water; D. Soak directly in warm water; E. Seal the package and soak in warm water; F. Put it in the refrigerator compartment in advance; G. Leave it at room temperature on a cupboard or cutting board	Scored item; safe thawing options were scored according to the original scoring scheme.
19	Preparation/kitchen hygiene	After handling raw meat, how do you usually wash your hands?<br>Options: A. Wipe with a towel or cloth; B. Wipe with a disposable wet wipe; C. Rinse with running cold tap water; D. Rinse with running warm tap water; E. Wash with soap and running cold tap water; F. Wash with soap and running warm tap water; G. Wash in a washbasin; H. Wash with soap in a washbasin; I. Usually do not wash	Scored item; washing with soap and running warm water was treated as the safer practice.
20	Preparation/kitchen hygiene	How do you usually clean a cutting board after cutting raw meat?<br>Options: A. Wipe it clean with a cloth; B. Scrape it with a knife and then wipe it clean; C. Rinse with running cold tap water; D. Rinse with running warm tap water; E. Rinse with running cold tap water and detergent; F. Rinse with running warm tap water and detergent; G. Do not clean it until the next use	Scored item; cleaning with running warm water and detergent was treated as the safer practice.
21	Preparation/cross-contamination	Do you usually use the same cutting board for raw meat and vegetables that will be eaten cold?<br>Options: A. Same board and same side; wash after cutting both; B. Same board but different sides; C. Same board and same side; rinse with running cold water after raw meat, then cut vegetables; D. Same board and same side; rinse with running warm water after raw meat, then cut vegetables; E. Same board and same side; clean with running cold water and detergent after raw meat, then cut vegetables; F. Same board and same side; clean with running warm water and detergent after raw meat, then cut vegetables; G. Two cutting boards are used; boards for raw meat and vegetables are not mixed	Scored item; using separate cutting boards was treated as the safer practice.

No.	Domain	English translation and response options	Scoring/status
22	Preparation/kitchen hygiene	Do you wash your hands before touching food?<br>Options: A. Always; if there is no way to wash hands, I do not touch food; B. Wash when conditions allow; otherwise do not wash; C. Wash when I remember; D. Rarely wash	Scored item; always washing hands was treated as the safer practice.
23	Preparation/kitchen hygiene	Which option best describes your handwashing habit?<br>Options: A. Wash with running cold tap water and dry; B. Wash with running warm tap water and dry; C. Wash carefully with running cold tap water and soap or hand sanitizer and dry; D. Wash carefully with running warm tap water and soap or hand sanitizer and dry; E. Wash in a basin with cold water and dry; F. Wash in a basin with warm water and dry; G. Wash carefully in a basin with cold water and soap or hand sanitizer and dry; H. Wash carefully in a basin with warm water and soap or hand sanitizer and dry	Scored item; using running warm water and soap/hand sanitizer was treated as the safer practice.
24	Leftover handling	How do you usually handle leftovers at home?<br>Options: A. Do not eat them; throw them away directly; B. Leave them directly in the cupboard; C. Seal them and leave them in the cupboard; D. Put them directly in the refrigerator compartment; E. Seal them and put them in the refrigerator compartment; F. Put them directly in the freezer; G. Seal them and put them in the freezer; H. There are never leftovers at home	Scored item; sealed cold storage was treated as the safer practice according to the original scoring scheme.
25	Leftover handling	Over how many meals are leftovers in your household usually eaten?<br>Options: A. Only one meal; B. Two to three meals; C. As long as they do not smell spoiled, keep eating until finished	Scored item; eating leftovers for only one meal was treated as the safer practice.
26	Leftover handling	How do you generally reheat leftovers?<br>Options: A. Heat until boiling; B. Heat until just warm enough to eat; it does not need to be too hot; C. Room temperature is enough; D. No need to heat in summer	Scored item; reheating until boiling was treated as the safer practice.
27	Foodborne disease risk	Which of the following situations do you think is most likely to cause food poisoning?<br>Options: A. Drinking yogurt just taken from the refrigerator; B. Eating raw or insufficiently heated seafood; C. Eating canned luncheon meat without heating; D. Eating fruit that has not been washed clean	Scored item; eating raw or insufficiently heated seafood was treated as the highest-risk option.

B. Household Food Safety Knowledge Section (36 scored items)

No.	Domain	English translation and response options	Scoring/status
1	Purchasing/food labels	Which type of food does not allow the use of genetic engineering technology during production?<br>Options: A. Organic food; B. Green food; C. Pollution-free food; D. Do not know	Correct answer scored 1; incorrect or do not know scored 0.
2	Purchasing/food labels	Which type of food uses reduced amounts of conventional pesticides and fertilizers during production?<br>Options: A. Organic food; B. Green food; C. Pollution-free food; D. Do not know	Correct answer scored 1; incorrect or do not know scored 0.

No.	Domain	English translation and response options	Scoring/status
3	Purchasing/food labels	Which type of food prohibits highly toxic and high-residue pesticides during production?<br>Options: A. Organic food; B. Green food; C. Pollution-free food; D. Do not know	Correct answer scored 1; incorrect or do not know scored 0.
4	Purchasing/transport	In summer, what is the safest way to take raw meat and seafood home after purchase?<br>Options: A. Use an ordinary bag and separate them from other foods; B. Put them together with other foods; C. Pack them separately with ice packs; D. It does not matter	Correct answer scored 1; incorrect or do not know scored 0.
5	Purchasing	When buying vegetables, fruits, raw meat, and seafood in a supermarket, what order do you think should be followed?<br>Options: A. No particular order; buy whatever you see; B. Buy raw meat and seafood first; C. Buy vegetables and fruits first, and raw meat and seafood later	Correct answer scored 1; incorrect scored 0.
6	Purchasing/food labels	Do you understand the meaning of the SC code for food production licensing in supermarkets?<br>Options: A. Understand; B. Do not understand; C. Have not heard of it	Correct answer scored 1; incorrect scored 0.
7	Purchasing/food labels	What determines the order of ingredients in a food ingredient list?<br>Options: A. Nutritional value; B. Purchase price; C. Amount/content of each ingredient; D. No order; determined by the manufacturer	Correct answer scored 1; incorrect scored 0.
8	Purchasing/food labels	Which of the following is not included in the nutrition facts table on food packaging?<br>Options: A. Energy; B. Trans fatty acids; C. Dietary fiber; D. Water	Correct answer scored 1; incorrect scored 0.
9	Purchasing/food additives	What are the main functions of food additives? (Multiple selection)<br>Options: A. Prevent food spoilage; B. Improve sensory properties; C. Maintain nutrition; D. Facilitate processing	All required correct options had to be selected and no incorrect option selected to score 1; otherwise 0.
10	Storage/food quality	Do you agree that “as long as food is within the shelf-life period, it is definitely safe to eat”?<br>Options: A. Disagree; B. Agree; C. It depends; even within shelf life, food should not be eaten if it seems abnormal	Correct answer scored 1; incorrect scored 0.
11	Storage	How should a large piece of raw meat just bought be stored?<br>Options: A. Put directly into the refrigerator; B. Cut into small pieces and put into the refrigerator; C. Cut into small pieces, seal them, and put into the refrigerator/freezer	Correct answer scored 1; incorrect scored 0.
12	Storage/freezing	Can freezing at -18°C kill bacteria in food?<br>Options: A. It can kill all bacteria; B. It can kill some bacteria; C. It cannot kill them completely	Correct answer scored 1; incorrect scored 0.
13	Purchasing/cold chain	During supermarket shopping, when is the best time to buy frozen foods?<br>Options: A. At the final stage of shopping; B. Anytime; it does not matter; C. Do not know	Correct answer scored 1; incorrect or do not know scored 0.

No.	Domain	English translation and response options	Scoring/status
14	Storage/freezing	What is the optimal freezer temperature?<br>Options: A. -8°C; B. -18°C; C. -4°C; D. Do not know	Correct answer scored 1; incorrect or do not know scored 0.
15	Storage/time-temperature control	If a meal has been prepared but will not be eaten until three hours later, what do you think is the best way to handle it?<br>Options: A. Put the food in the refrigerator and reheat it before eating; B. Keep it warm in a microwave oven; C. Cover it and leave it in the cupboard; D. Put it in the cupboard and reheat before eating	Correct answer scored 1; incorrect scored 0.
16	Storage and thawing	Can thawed meat be frozen again?<br>Options: A. Yes; B. No; C. It does not matter; if it cannot be eaten, freeze it again	Correct answer scored 1; incorrect scored 0.
17	Preparation/produce washing	How do you think vegetables and fruits should be washed?<br>Options: A. Wash with detergent after soaking in water; B. Wash with hot water; C. Wash under running cold water; D. Soak in cold water and wash	Correct answer scored 1; incorrect scored 0.
18	Storage and thawing	Which of the following methods for thawing raw meat do you think is the most unsafe?<br>Options: A. Thawing in the refrigerator compartment; B. Thawing slowly on a cutting board; C. Thawing in a microwave oven; D. Thawing in cold water after sealing tightly	Correct answer scored 1; incorrect scored 0.
19	Leftover handling	How should leftovers be reheated?<br>Options: A. Reheat according to personal preference; B. No need to reheat in summer; eat directly; C. Reheat until fully boiling	Correct answer scored 1; incorrect scored 0.
20	Leftover handling	What is the correct way to handle leftovers?<br>Options: A. As long as they are not spoiled, they can be eaten without reheating; B. Put them in the refrigerator immediately and reheat thoroughly before the next consumption; C. Leave them in a cupboard and reheat thoroughly next time; D. Throw them away immediately	Correct answer scored 1; incorrect scored 0.
21	Storage/cross-contamination	If the refrigerator compartment has three shelves, on which shelf should raw meat be placed?<br>Options: A. Top shelf; B. Middle shelf; C. Bottom shelf	Correct answer scored 1; incorrect scored 0.
22	Storage/refrigeration	What is the appropriate temperature for the refrigerator compartment?<br>Options: A. 5°C; B. 4°C; C. 0°C; D. Do not know	Correct answer scored 1; incorrect or do not know scored 0.
23	Leftover handling	What is the maximum length of time leftovers should be kept in the refrigerator?<br>Options: A. Within two days; B. Within five days; C. As long as they are not spoiled, they can be kept for any length of time	Correct answer scored 1; incorrect scored 0.
24	Kitchen hygiene	What is the correct way to clean kitchen countertops or stovetops?<br>Options: A. Wipe clean with a dry cloth; B. Wipe clean with a wet cloth; C. Clean thoroughly with warm water and detergent	Correct answer scored 1; incorrect scored 0.

No.	Domain	English translation and response options	Scoring/status
25	Kitchen hygiene	Which of the following do you think are correct ways to wash dishes? (Multiple selection)<br>Options: A. Use an automatic dishwasher; B. Soak in water for several hours and then wash with the soaking water; C. Wash dishes immediately after meals and air-dry naturally; D. Wash in a basin and then dry with a dish towel; E. Other method	All required correct options had to be selected and no incorrect option selected to score 1; otherwise 0.
26	Kitchen hygiene/cross-contamination	After a cutting board has been used to cut meat, how can it be made safe and hygienic before cutting fruit? (Multiple selection)<br>Options: A. Wipe the cutting board clean with a cloth; B. Rinse the cutting board clean with hot water; C. Use the other side of the cutting board to cut fruit; D. Clean the cutting board with hot water and detergent before cutting fruit; E. Use another cutting board to cut fruit; F. Other method	All required correct options had to be selected and no incorrect option selected to score 1; otherwise 0.
27	Preparation/food handler hygiene	If there is a wound on your hand, do you think it is safe to handle food?<br>Options: A. It is safe as long as the wound is not infected; B. It is fine if the wound is covered with a bandage; C. It is acceptable if gloves are worn; D. All of the above	Correct answer scored 1; incorrect scored 0.
28	Preparation/handwashing	What is the correct handwashing method?<br>Options: A. Rinse clean with running tap water and dry; B. Rinse clean with running warm water and dry; C. Wash hands in a basin and dry; D. Wet hands with basin water, apply soap, wash with basin water, and dry; E. Wet hands with running warm water, apply soap, rinse clean with running warm water, and dry	Correct answer scored 1; incorrect scored 0.
29	Preparation/handwashing	After handling raw meat, how should hands be washed?<br>Options: A. Wipe hands with a towel; B. Wash hands with cold water; C. Wash hands with warm water; D. Wash hands with warm water and soap; E. Wipe hands with a wet wipe	Correct answer scored 1; incorrect scored 0.
30	Preparation/handwashing	When preparing food, after touching which of the following should hands be washed? (Multiple selection)<br>Options: A. Face; B. Fresh fruit; C. Pimples or spots on the skin; D. Clothes; E. Clean bowls and plates; F. Other	All required correct options had to be selected and no incorrect option selected to score 1; otherwise 0.
31	Preparation/food handler illness	Under which of the following conditions do you think a person is not suitable for cooking? (Multiple selection)<br>Options: A. Diarrhea; B. Skin allergy; C. High fever; D. Sore throat; E. Cold; F. AIDS; G. Other	All required correct options had to be selected and no incorrect option selected to score 1; otherwise 0.
32	Foodborne disease prevention	Which do you think is the most critical for preventing food poisoning?<br>Options: A. Spray the kitchen with pesticide weekly; B. Avoid eating leftovers; C. Store food in the refrigerator before eating; D. Clean kitchen countertops or stoves weekly with detergent	Correct answer scored 1; incorrect scored 0.
33	Foodborne pathogens	Which of the following do you think can prevent Salmonella poisoning?<br>Options: A. Cook food thoroughly; B. Wash food with hot water; C. Freeze food for more than three days; D. Do not know	Correct answer scored 1; incorrect or do not know scored 0.

No.	Domain	English translation and response options	Scoring/status
34	Foodborne pathogens	Which of the following is most likely to be contaminated with Escherichia coli? Options: A. River water; B. Raw pork; C. Raw beef; D. Raw eggs; E. Do not know	Correct answer scored 1; incorrect or do not know scored 0.
35	Foodborne pathogens	Which of the following is most likely to be contaminated with Listeria? Options: A. Raw meat; B. Cooked deli foods bought from a deli; C. Raw eggs; D. Do not know	Correct answer scored 1; incorrect or do not know scored 0.
36	Foodborne disease risk	Which of the following options can cause food poisoning? Options: A. Fruit just taken from the refrigerator; B. Unheated canned food; C. Raw eggs; D. Undercooked beef; E. Options C and D; F. Other	Correct answer scored 1; incorrect scored 0.

C. Demographic Questions from the Field Questionnaire (not counted in outcome item totals)

No.	English item	Response options
1	Age	A. 18-24; B. 25-34; C. 35-44; D. 45-54; E. 55-64; F. 65 years or above
2	Sex	A. Male; B. Female
3	Highest educational level	A. Below primary school; B. Primary school; C. Junior high school; D. Senior high school or technical secondary school; E. College, university, or above
4	Current marital status	A. Unmarried; B. Married; C. Other
5	Average daily working hours	A. 0-1 hour; B. 2-3 hours; C. 4-5 hours; D. 6-7 hours; E. 8 hours or more
6	Household annual income last year	A. Below 12,000 CNY; B. 12,000-36,000 CNY; C. 36,000-60,000 CNY; D. 60,000-84,000 CNY; E. Above 84,000 CNY
7	Number of permanent household members	Open-ended
8	Does your household have children under 6 years old?	A. No; B. Yes
9	Does your household have adults aged 65 years or above?	A. No; B. Yes
10	Current health condition	A. Healthy with no disease; B. Hypertension; C. Diabetes; D. Hyperlipidemia; E. Other disease; F. Physical discomfort but unclear disease
